# Supplementary material for: Estrogen mediates sex differences in preoptic neuropeptide and pituitary hormone production in medaka
Source: Commun Biol. 2021 Aug 9;4:948. doi: 10.1038/s42003-021-02476-5 (PMC8352984; doi:10.1038/s42003-021-02476-5)
Supplement: Supplementary file 1 — Reporting Summary [file 42003_2021_2476_MOESM1_ESM.pdf]

nature portfolio

Corresponding author(s): Kataaki Okubo

Last updated by author(s): Jul 11, 2021

Reporting Summary

Nature Portfolio wishes to improve the reproducibility of the work that we publish. This form provides structure for consistency and transparency in reporting. For further information on Nature Portfolio policies, see our Editorial Policies and the Editorial Policy Checklist.

Statistics

For all statistical analyses, confirm that the following items are present in the figure legend, table legend, main text, or Methods section.

n/a	Confirmed	
☐	☒	The exact sample size (*n*) for each experimental group/condition, given as a discrete number and unit of measurement
☐	☒	A statement on whether measurements were taken from distinct samples or whether the same sample was measured repeatedly
☐	☒	The statistical test(s) used AND whether they are one- or two-sided<br>*Only common tests should be described solely by name; describe more complex techniques in the Methods section.*
☒	☐	A description of all covariates tested
☐	☒	A description of any assumptions or corrections, such as tests of normality and adjustment for multiple comparisons
☐	☒	A full description of the statistical parameters including central tendency (e.g. means) or other basic estimates (e.g. regression coefficient) AND variation (e.g. standard deviation) or associated estimates of uncertainty (e.g. confidence intervals)
☐	☒	For null hypothesis testing, the test statistic (e.g. *F*, *t*, *r*) with confidence intervals, effect sizes, degrees of freedom and *P* value noted<br>*Give P values as exact values whenever suitable.*
☒	☐	For Bayesian analysis, information on the choice of priors and Markov chain Monte Carlo settings
☒	☐	For hierarchical and complex designs, identification of the appropriate level for tests and full reporting of outcomes
☒	☐	Estimates of effect sizes (e.g. Cohen's *d*, Pearson's *r*), indicating how they were calculated

Our web collection on statistics for biologists contains articles on many of the points above.

Software and code

Policy information about availability of computer code

Data collection	ClustalW (http://clustalw.ddbj.nig.ac.jp/index.php)
Data analysis	Adobe Photoshop (ver. 22); ImageJ (http://rsbweb.nih.gov/ij/); GraphPad Prism (ver. 8)

For manuscripts utilizing custom algorithms or software that are central to the research but not yet described in published literature, software must be made available to editors and reviewers. We strongly encourage code deposition in a community repository (e.g. GitHub). See the Nature Portfolio guidelines for submitting code & software for further information.

Data

Policy information about availability of data

All manuscripts must include a data availability statement. This statement should provide the following information, where applicable:

- Accession codes, unique identifiers, or web links for publicly available datasets
- A description of any restrictions on data availability
- For clinical datasets or third party data, please ensure that the statement adheres to our policy

The medaka adcyap1 and vip cDNA sequences have been deposited in GenBank under accession numbers LC579549 and LC579550, respectively. All other data supporting the findings of this study are available within the article and its supplementary information or from the corresponding author upon reasonable request. The original uncropped images of Northern blots are shown in Supplementary Fig. 6.

Field-specific reporting

Please select the one below that is the best fit for your research. If you are not sure, read the appropriate sections before making your selection.

[x] Life sciences [ ] Behavioural & social sciences [ ] Ecological, evolutionary & environmental sciences

For a reference copy of the document with all sections, see nature.com/documents/nr-reporting-summary-flat.pdf

Life sciences study design

All studies must disclose on these points even when the disclosure is negative.

Sample size	Power analysis was not performed prior to beginning the study due to the lack of existing data on medaka adcyap1 and vip neurons. The sample size was estimated on the basis of our previous studies which provided information on inter-individual variation in neural gene expression levels in medaka (e.g., Hiraki-Kajiyama et al., 2019, eLife, 8:e39495; Yamashita et al., 2020, eLife, e59470; Nishiike et al., 2021, Curr Biol, 31:1699-1710).
Data exclusions	No test for outliers was performed and all data were included in the statistical analyses.
Replication	Since the analysis on different individuals yielded similar results, we believe that the results are reproducible.
Randomization	Animals were randomly assigned to experimental groups.
Blinding	Blinding was not performed because most of the data collection and analysis was done by automated software.

Reporting for specific materials, systems and methods

We require information from authors about some types of materials, experimental systems and methods used in many studies. Here, indicate whether each material, system or method listed is relevant to your study. If you are not sure if a list item applies to your research, read the appropriate section before selecting a response.

Materials & experimental systems

n/a	Involved in the study	
[ ]	[x]	Antibodies
[ ]	[x]	Eukaryotic cell lines
[x]	[ ]	Palaeontology and archaeology
[ ]	[x]	Animals and other organisms
[x]	[ ]	Human research participants
[x]	[ ]	Clinical data
[x]	[ ]	Dual use research of concern

Methods

n/a	Involved in the study	
[x]	[ ]	ChIP-seq
[x]	[ ]	Flow cytometry
[x]	[ ]	MRI-based neuroimaging

Antibodies

Antibodies used	Anti-PACAP antibody (RRID: AB_519166; Peninsula Laboratories); Anti-VIP antibody (RRID: AB_572270; ImmunoStar)
Validation	These antibodies have been shown to recognize teleost Pacap and Vip, respectively, with high specificity (e.g., Olsson and Holmgren, 1994, doi:10.1007/BF00300227; Wong et al., 1998, doi:10.1210/endo.139.8.6145; Finney et al., 2006, doi:10.1002/cne.20948; Uyttebroek et al., 2010, doi:10.1002/cne.22464; Uyttebroek et al., 2013, doi:10.1007/s00441-013-1685-8). The specificity of these antibodies was further verified by preabsorbing with the synthetic medaka Pacap and Vip polypeptides, which blocked immunodetection.

Eukaryotic cell lines

Policy information about cell lines

Cell line source(s)	COS-7 cells: Riken BRC Cell Bank
Authentication	Cells were authenticated by the supplier (Riken BRC Cell Bank).
Mycoplasma contamination	Cells were confirmed to be mycoplasma free by the supplier (Riken BRC Cell Bank).
Commonly misidentified lines (See ICLAC register)	n/a

Animals and other organisms

Policy information about studies involving animals; ARRIVE guidelines recommended for reporting animal research

Laboratory animals	Species: Medaka (Oryzias latipes)<br>Strain: d-rR<br>Sex: Fish of both sexes were used in all analyses.<br>Age: Spawning adult fish (aged 2–4 months) were used in all analyses except for the determination of gene expression levels during growth and sexual maturation, where fish aged 1, 2, 3, and 7 months were used.
Wild animals	n/a
Field-collected samples	n/a
Ethics oversight	n/a

Note that full information on the approval of the study protocol must also be provided in the manuscript.
