# Supplementary material for: Estrogen mediates sex differences in preoptic neuropeptide and pituitary hormone production in medaka
Source: Commun Biol. 2021 Aug 9;4:948. doi: 10.1038/s42003-021-02476-5 (PMC8352984; doi:10.1038/s42003-021-02476-5)
Supplement: Supplementary file 2 — Supplementary Information [file 42003_2021_2476_MOESM2_ESM.pdf]

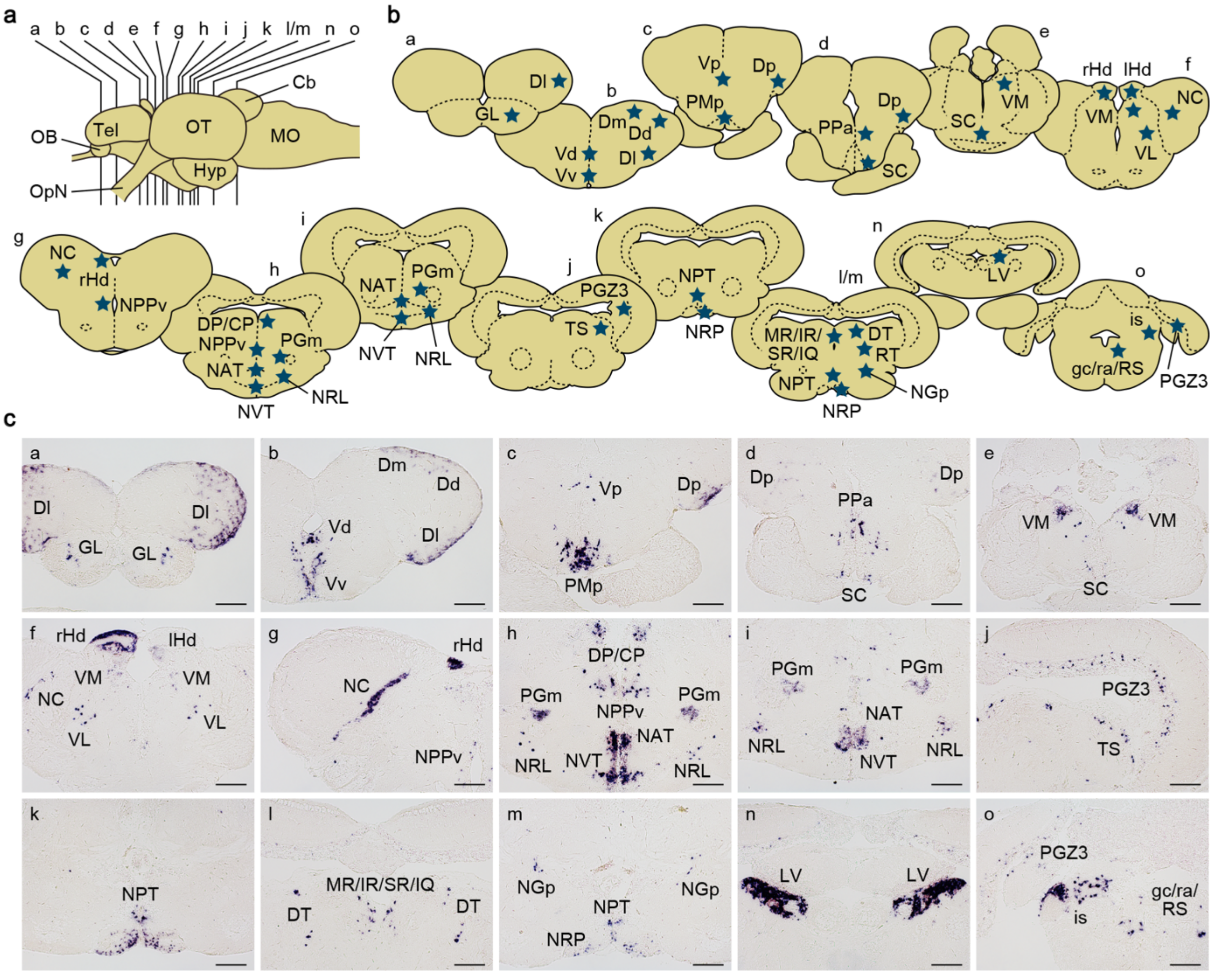

Supplementary Fig. 1: Distribution of *adcyap1*-expressing neurons in the medaka brain. **a** Lateral view (anterior to the left) of the medaka brain showing the approximate levels of sections in panels **b** and **c**. **b** Line drawings of coronal brain sections showing the location of nuclei containing *adcyap1*-expressing neurons (stars). **c** Representative micrographs of coronal brain sections showing the distribution of *adcyap1*-expressing neurons. Scale bars represent 100 µm. For abbreviations of brain regions and nuclei, see Supplementary Table 1.

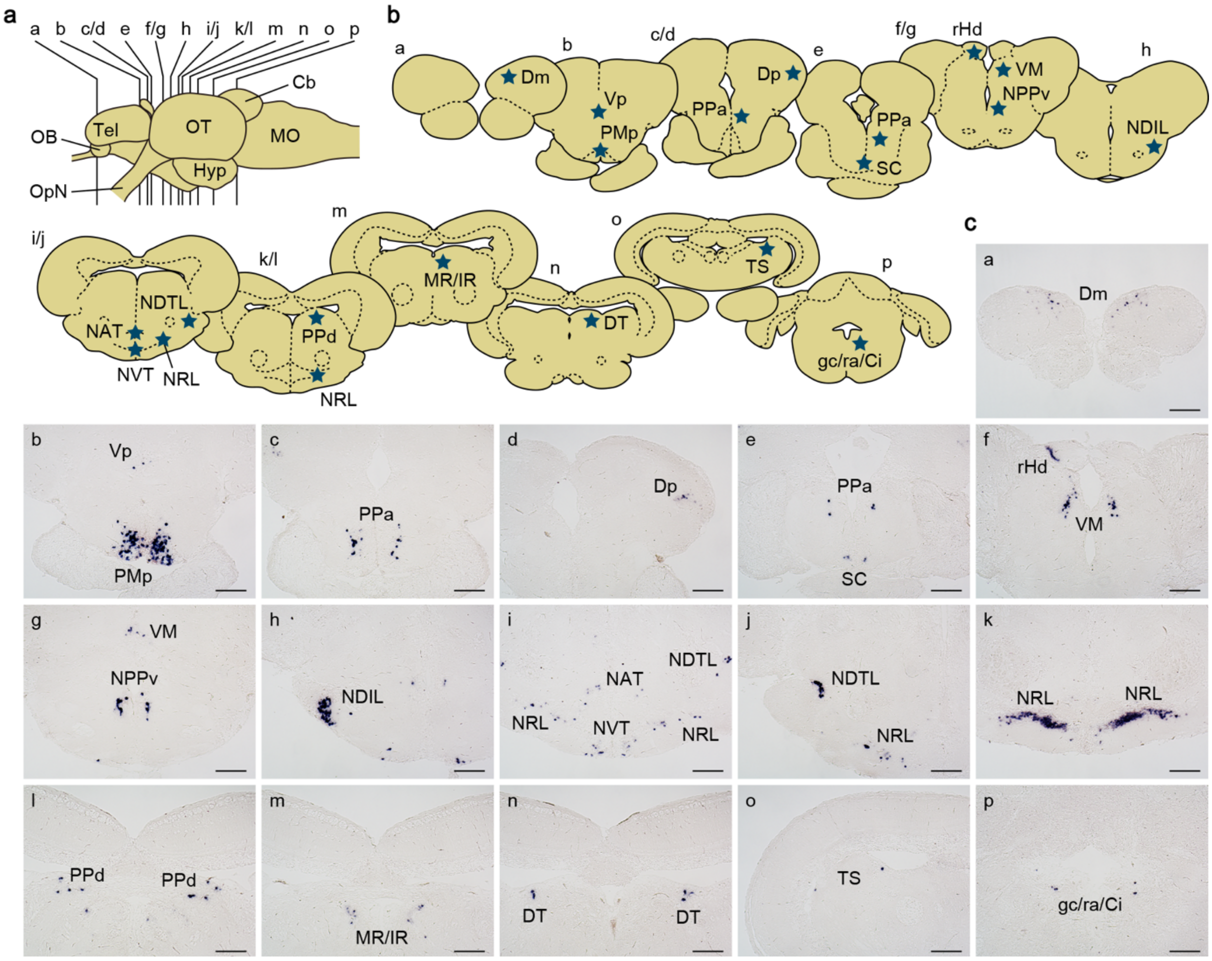

Supplementary Fig. 2: Distribution of *vip*-expressing neurons in the medaka brain.
a Lateral view (anterior to the left) of the medaka brain showing the approximate levels of sections in panels **b** and **c**. **b** Line drawings of coronal brain sections showing the location of nuclei containing *vip*-expressing neurons (stars). **c** Representative micrographs of coronal brain sections showing the distribution of *vip*-expressing neurons. Scale bars represent 100 µm. For abbreviations of brain regions and nuclei, see Supplementary Table 1.

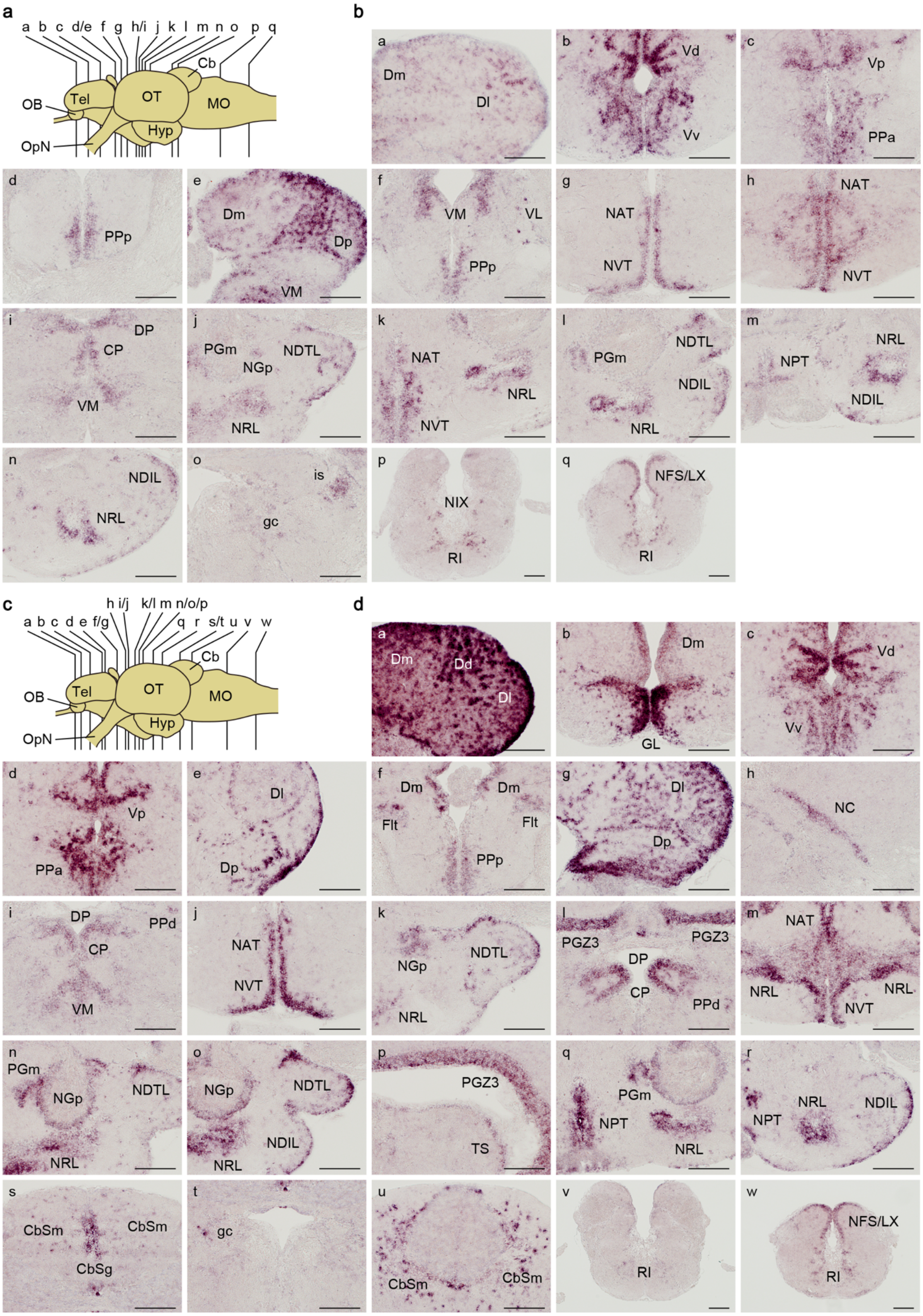

Supplementary Fig. 3: Distribution of *adcyap1r1a* and *adcyap1r1b* expression in the medaka brain.

a Lateral view (anterior to the left) of the medaka brain showing the approximate levels of sections in

panel **b**. **b** Representative micrographs of coronal brain sections showing the distribution of *adcyap1r1a* expression. **c** Lateral view of the medaka brain showing the approximate levels of sections in panel **d**. **d** Representative micrographs of coronal brain sections showing the distribution of *adcyap1r1b* expression. Scale bars represent 100 μm. For abbreviations of brain regions and nuclei, see Supplementary Table 1.

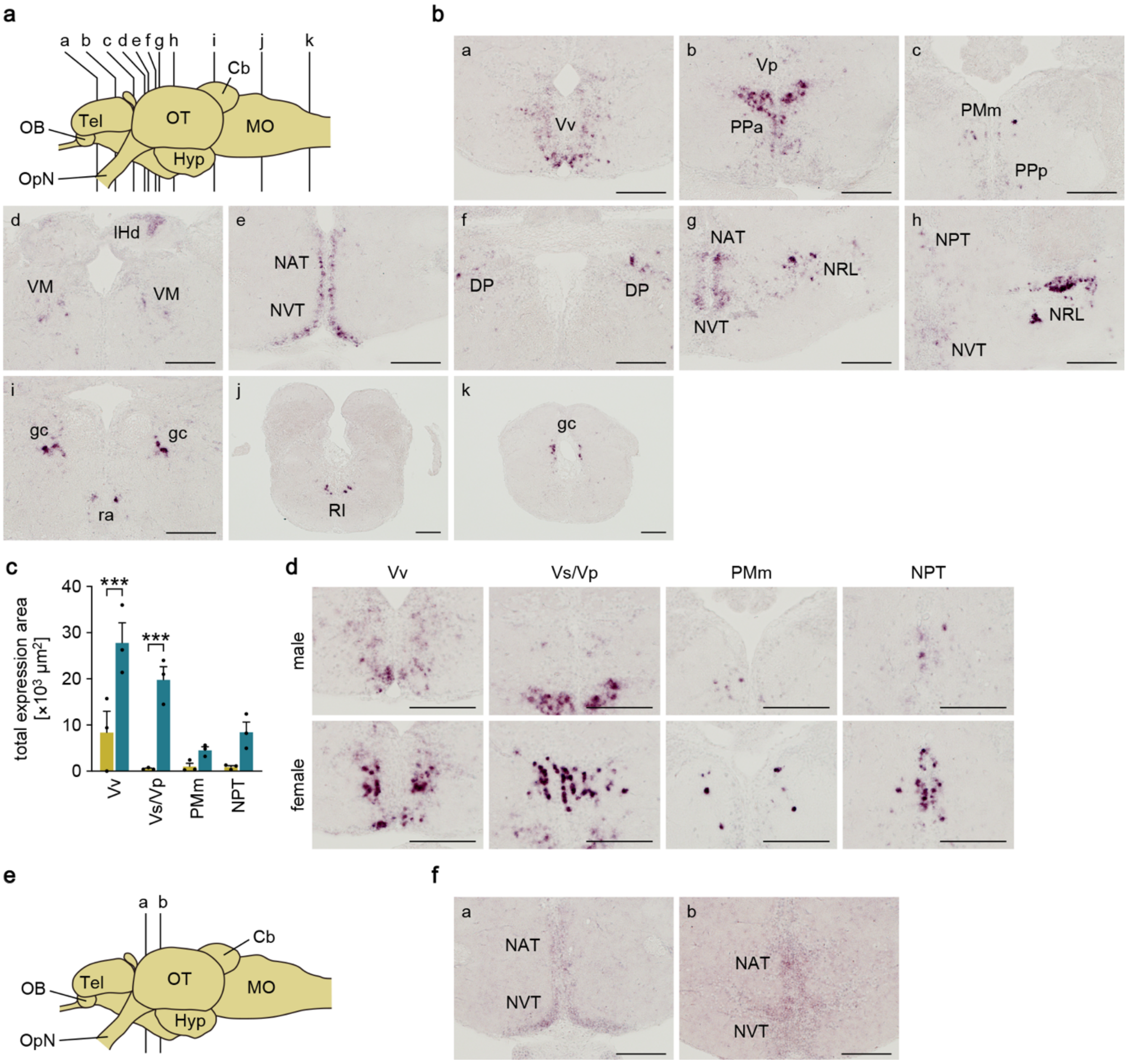

Supplementary Fig. 4: Distribution of *vipr1a* and *vipr1b* expression in the medaka brain. **a** Lateral view (anterior to the left) of the medaka brain showing the approximate levels of sections in panel **b**. **b** Representative micrographs of coronal brain sections showing the distribution of *vipr1a* expression. **c** Total area of *vipr1a* expression signals in the Vv, Vs/Vp, PMm, and NPT nuclei of males and females ($n = 3$ per sex). **d** Representative micrographs showing *vipr1a* expression in the Vv, Vs/Vp, PMm, and NPT nuclei of males and females. **e** Lateral view of the medaka brain showing the approximate levels of sections in panel **f**. **f** Representative micrographs of coronal brain sections showing the distribution of *vipr1b* expression. Scale bars represent 100 µm. Quantitative data were expressed as means with error bars representing standard error of the mean. Statistical differences were assessed by unpaired *t*-test with Bonferroni-Dunn correction (**c**). ***$p < 0.001$. For abbreviations of brain regions and nuclei, see Supplementary Table 1.

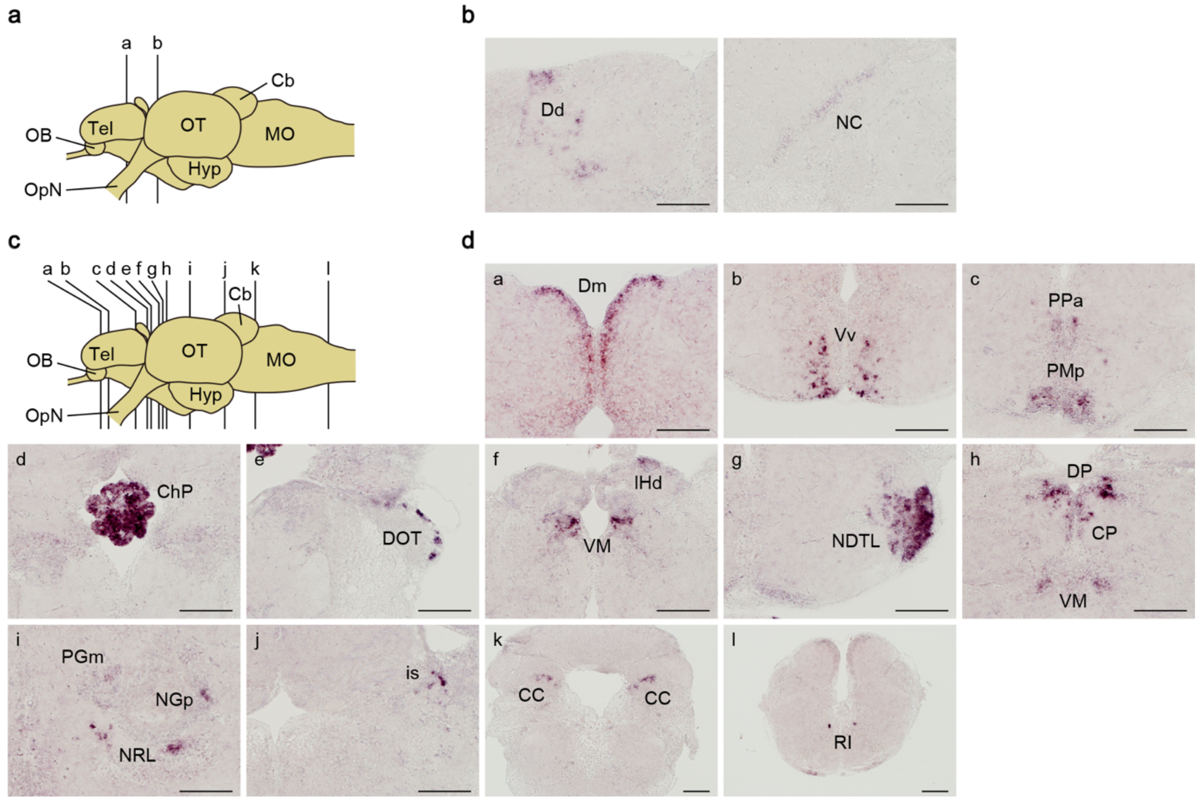

Supplementary Fig. 5: Distribution of *vipr2a* and *vipr2b* expression in the medaka brain. **a** Lateral view (anterior to the left) of the medaka brain showing the approximate levels of sections in panel **b**. **b** Representative micrographs of coronal brain sections showing the distribution of *vipr2a* expression. **c** Lateral view of the medaka brain showing the approximate levels of sections in panel **d**. **d** Representative micrographs of coronal brain sections showing the distribution of *vipr2b* expression. Scale bars represent 100 μm. For abbreviations of brain regions and nuclei, see Supplementary Table 1.

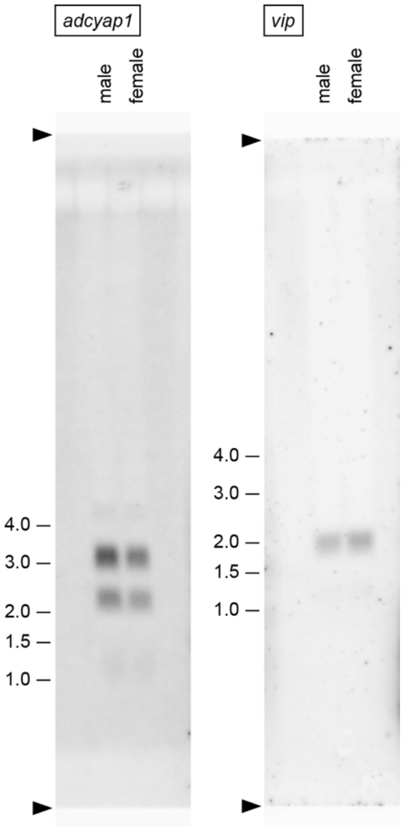

Supplementary Fig. 6: Original uncropped images of Northern blots.
Sizes (in kb) of RNA markers are indicated on the left. The top and bottom edges of the gels/membranes are indicated by arrowheads.

Supplementary Table 1: Abbreviations of brain regions and brain nuclei.

abbreviation	full name	location
brain regions		
Cb	cerebellum	
ChP	choroid plexus	
Hyp	hypothalamus	
MO	medulla oblongata	
OB	olfactory bulb	
OpN	optic nerve	
OT	optic tectum	
Tel	telencephalon	
brain nucleus		
CbSg	granular layer of cerebellum	cerebellum
CbSm	molecular layer of cerebellum	cerebellum
CC	cerebellar crest	brain stem
Ci	interpeduncular corpus	brain stem
CP	central posterior nucleus	thalamus
Dd	dorsal nucleus of the dorsal telencephalic area	dorsal telencephalon
Dl	lateral nucleus of the dorsal telencephalic area	dorsal telencephalon
Dm	medial nucleus of the dorsal telencephalic area	dorsal telencephalon
Dp	posterior nucleus of the dorsal telencephalic area	dorsal telencephalon
DP	dorsal posterior nucleus	thalamus
DT	dorsal tegmental nucleus	midbrain tegmentum
Flt	telencephalic lateral longitudinal fascicle	thalamus
gc	central gray	brain stem
GL	glomerular layer of the olfactory bulb	olfactory bulb
IQ	inferior oblique of the oculomotor nerve nucleus	midbrain tegmentum
IR	inferior rectus of the oculomotor nerve nucleus	midbrain tegmentum
is	isthmus nucleus	midbrain tegmentum
lHd	left dorsal habenula	habenula
LV	lateral valvular nucleus	midbrain tegmentum
MR	medial rectus of the oculomotor nerve nucleus	midbrain tegmentum
NAT	anterior tuberal nucleus	hypothalamus
NC	cortical nucleus	pretectum
NDIL	diffuse nucleus of inferior lobe	hypothalamus
NDTL	diffuse nucleus of lateral torus	hypothalamus
NFS/LX	nucleus of solitary fascicle/vagal lobe	brain stem
NGp	posterior part of the glomerular nucleus	hypothalamus
NIX	nucleus of glossopharyngeal nerve	brain stem
NPPv	posterior periventricular nucleus	hypothalamus
NPT	posterior tuberal nucleus	hypothalamus
NRL	lateral recess nucleus	hypothalamus
NRP	posterior recess nucleus	hypothalamus
NVT	ventral tuberal nucleus	hypothalamus
PGm	medial preglomerular nucleus	hypothalamus
PGZ3	periventricular gray zone (layer 3)	optic tectum
PMm	magnocellular portion of the magnocellular preoptic nucleus	preoptic area
PMp	parvocellular portion of the magnocellular preoptic nucleus	preoptic area
PPa	anterior parvocellular preoptic nucleus	preoptic area

PPd	dorsal periventricular pretectal nucleus	pretectum
PPp	posterior parvocellular preoptic nucleus	preoptic area
ra	raphe nucleus	brain stem
rHd	right dorsal habenula	habenula
RI	inferior reticular nucleus	brain stem
RS	superior reticular nucleus	brain stem
RT	rostral tegmental nucleus	midbrain tegmentum
SC	suprachiasmatic nucleus	preoptic area
SR	superior rectus of the oculomotor nerve nucleus	midbrain tegmentum
TS	semicircular torus	midbrain tegmentum
VL	ventrolateral nucleus	thalamus
VM	ventromedial nucleus	thalamus
Vd	dorsal nucleus of the ventral telencephalic area	ventral telencephalon
Vp	posterior nucleus of the ventral telencephalic area	ventral telencephalon
Vs	supracommissural nucleus of the ventral telencephalic area	ventral telencephalon
Vv	ventral nucleus of the ventral telencephalic area	ventral telencephalon

Supplementary Table 2: Species names and GenBank accession numbers of the protein sequences used in this study.

protein	species	accession number
ADCYAP1	human (*Homo sapiens*)	NP_001093203
ADCYAP1	mouse (*Mus musculus*)	NP_033755
Adcyap1	chicken (*Gallus gallus*)	NP_001001291
Adcyap1	*Xenopus laevis*	NP_001081947
Adcyap1	medaka (*Oryzias latipes*)	LC579549
Adcyap1a	fugu (*Takifugu rubripes*)	NP_001106662
Adcyap1a	zebrafish (*Danio rerio*)	NP_690841
Adcyap1b	fugu (*Takifugu rubripes*)	NP_001106663
Adcyap1b	zebrafish (*Danio rerio*)	NP_999880
VIP	human (*Homo sapiens*)	NP_003372
VIP	mouse (*Mus musculus*)	NP_035832
Vip	chicken (*Gallus gallus*)	NP_990697
Vip	*Xenopus laevis*	NP_001079183
Vip	fugu (*Takifugu rubripes*)	NP_001106661
Vip	medaka (*Oryzias latipes*)	LC579550
Vip	zebrafish (*Danio rerio*)	NP_001108025
SCT	human (*Homo sapiens*)	NP_068739
SCT	mouse (*Mus musculus*)	NP_001274100
ADCYAP1R1	human (*Homo sapiens*)	NP_001186564
ADCYAP1R1	mouse (*Mus musculus*)	NP_031433
Adcyap1r1	chicken (*Gallus gallus*)	NP_001092076
Adcyap1r1a	fugu (*Takifugu rubripes*)	NP_001098685
Adcyap1r1a	medaka (*Oryzias latipes*)	XP_023820892
Adcyap1r1a	zebrafish (*Danio rerio*)	NP_001136397
Adcyap1r1b	fugu (*Takifugu rubripes*)	NP_001098686
Adcyap1r1b	medaka (*Oryzias latipes*)	XP_004068178
VIPR1	human (*Homo sapiens*)	NP_004615
VIPR1	mouse (*Mus musculus*)	NP_035833
Vipr1	chicken (*Gallus gallus*)	NP_001090992
Vipr1	zebrafish (*Danio rerio*)	NP_001013371
Vipr1a	fugu (*Takifugu rubripes*)	CAC82588
Vipr1a	medaka (*Oryzias latipes*)	XP_023805948
Vipr1b	fugu (*Takifugu rubripes*)	CAC82587
Vipr1b	medaka (*Oryzias latipes*)	XP_004079154
VIPR2	human (*Homo sapiens*)	NP_003373
VIPR2	mouse (*Mus musculus*)	NP_033537
Vipr2	chicken (*Gallus gallus*)	NP_001014970
Vipr2	zebrafish (*Danio rerio*)	NP_571854
Vipr2a (partial sequence)	fugu (*Takifugu rubripes*)	CAC83860
Vipr2a	medaka (*Oryzias latipes*)	XP_023805816
Vipr2b (partial sequence)	fugu (*Takifugu rubripes*)	CAC83861
Vipr2b	medaka (*Oryzias latipes*)	XP_023821167
SCTR	human (*Homo sapiens*)	NP_002971
SCTR	mouse (*Mus musculus*)	NP_001012322

Supplementary Table 3: Primers used for real-time PCR.

target	direction	sequence (5´–3´)
adcyap1	forward	TGTCCTACAAGAGCTAGA
adcyap1	reverse	CGTGGAAAGAGCCAGAAA
vip	forward	TCAGACGCCATCTTCACA
vip	reverse	CAGGTACTTCTTGACTGCCATC
fshb	forward	GACTGGTCCTACGAAGTTA
fshb	reverse	TGTGGTTCTTGTGTTGCATGT
lhb	forward	GTGGATCCGTCAGTCACATACC
lhb	reverse	GTGCAGTCAGACGCGTTCAT
tshb	forward	TTACCTACCCCGTGGCACTC
tshb	reverse	TGCGTGCACTCATCACTGTC
gh	forward	CTTTTCTCTGACTTTGAGAGTT
gh	reverse	GTGCTTGTCTAATGGGCTGATG
sl	forward	GCATCACCAAAGCATTACC
sl	reverse	ATGCAGCAGCCATTTATCAGA
prl	forward	TCCTGTCCAACTCTGCAAACTC
prl	reverse	CAGGTTCCTGGAATGCTCCT
pomc	forward	AGCAGCATGACGGAGT
pomc	reverse	GGAGAGATGAAAGAGAAGGGA
actb	forward	CCCCACCCAAAGTTTAG
actb	reverse	CAACGATGGAGGGAAAGACA
gapdh	forward	GACCTCCATGTTGGAATCAATG
gapdh	reverse	AATGAAGGGGTCGTTGATGG
rpl13	forward	ACTCATCCTGTTCCCAAGGAAG
rpl13	reverse	CCACTGAGCTGAGTAGCCATCTT
