# Supplementary material for: Estrogen mediates sex differences in preoptic neuropeptide and pituitary hormone production in medaka
Source: Commun Biol. 2021 Aug 9;4:948. doi: 10.1038/s42003-021-02476-5 (PMC8352984; doi:10.1038/s42003-021-02476-5)
Supplement: Supplementary file 4 — Description of Supplementary Files [file 42003_2021_2476_MOESM4_ESM.pdf]

Description of Additional Supplementary Files

File name: Supplementary Data 1
Description: Source data for all figures.
